# The Efficacy of Health Information Technology in Supporting Health Equity for Black and Hispanic Patients With Chronic Diseases: Systematic Review

**DOI:** 10.2196/22124

**Published:** 2022-04-04

**Authors:** Charles Senteio, Paul Joseph Murdock

**Affiliations:** 1 Department of Library and Information Science School of Communication and Information Rutgers University New Brunswick, NJ United States; 2 Division of Health Sciences Informatics School of Medicine Johns Hopkins University Baltimore, MD United States

**Keywords:** chronic disease, minority health, technology assessment, biomedical, self-management, systematic review, mobile phone

## Abstract

**Background:**

Racial inequity persists for chronic disease outcomes amid the proliferation of health information technology (HIT) designed to support patients in following recommended chronic disease self-management behaviors (ie, medication behavior, physical activity, and dietary behavior and attending follow-up appointments). Numerous interventions that use consumer-oriented HIT to support self-management have been evaluated, and some of the related literature has focused on racial minorities who experience disparate chronic disease outcomes. However, little is known about the efficacy of these interventions.

**Objective:**

This study aims to conduct a systematic review of the literature that describes the efficacy of consumer-oriented HIT interventions designed to support self-management involving African American and Hispanic patients with chronic diseases.

**Methods:**

We followed an a priori protocol using the PRISMA (Preferred Reporting Items for Systematic Reviews and Meta-Analyses)-Equity 2012 Extension guidelines for systematic reviews that focus on health equity. Themes of interest included the inclusion and exclusion criteria. We identified 7 electronic databases, created search strings, and conducted the searches. We initially screened results based on titles and abstracts and then performed full-text screening. We then resolved conflicts and extracted relevant data from the included articles.

**Results:**

In total, there were 27 included articles. The mean sample size was 640 (SD 209.5), and 52% (14/27) of the articles focused on African American participants, 15% (4/27) of the articles focused on Hispanic participants, and 33% (9/27) included both. Most articles addressed 3 of the 4 self-management behaviors: medication (17/27, 63%), physical activity (17/27, 63%), and diet (16/27, 59%). Only 15% (4/27) of the studies focused on follow-up appointment attendance. All the articles investigated HIT for use at home, whereas 7% (2/27) included use in the hospital.

**Conclusions:**

This study addresses a key gap in research that has not sufficiently examined what technology designs and capabilities may be effective for underserved populations in promoting health behavior in concordance with recommendations.

## Introduction

### Background

Nearly half of all adults in the United States are living with 1 or more of the *Big Five* chronic conditions—diabetes mellitus (*diabetes*), cardiovascular disease, chronic respiratory disease, cancer, and stroke [1]. Racial inequity persists for outcomes under these conditions [2]. For example, African American individuals continue to experience greater disease prevalence than non-Hispanic White individuals for hypertension (25%) and diabetes (49%); likewise, Hispanic individuals’ diabetes rates are 20% higher than those of White individuals [3]. Furthermore, nearly 5 decades of literature details racial and ethnic inequity in diabetes prevalence and risk factors for diabetes-related complications and following recommended self-management behavior [4].

Chronic disease self-management is challenging because the treatment regimens often demand much from the patient and their families; recommended self-management frequently includes regular meal planning, consistent physical activity, monitoring and tracking (eg, fluid intake and blood glucose), and daily medication behavior [5]. Following the recommended self-management behavior is vital because these behaviors are associated with health outcomes. For example, following the recommended medication behavior, physical activity, dietary behavior, and blood sugar testing are all associated with glycemic control [6]. Comorbidity can exacerbate the burden associated with following self-management recommendations. For example, a cancer survivor with diabetes who must take medication as part of their cancer treatment (eg, prednisone) may experience difficulty in maintaining the recommended glucose levels, which can in turn impact medication behavior [7]. Chemotherapy can also cause adverse side effects, including pain and cognitive impairment. Both can present barriers to following recommended self-management for years following cancer treatment [8], and cancer survivors from racial minority groups experience poorer outcomes for other chronic conditions diagnosed after a cancer diagnosis [9].

Patients with chronic diseases may use information technology (eg, mobile apps) as sources of health information to help answer questions regarding symptoms and treatment options [10-12]. However, racial inequity also characterizes access to information and communication technologies (ICTs). Most White individuals own a laptop or desktop computer (83%), whereas only about two-thirds of African American individuals (66%) reported owning either. There is also racial and ethnic inequity in access to broadband at home, with 78% of the White population reporting access compared with 65% of African American individuals and 58% of Hispanic individuals [13]. African American individuals and Hispanic individuals own smartphones and tablets at similar rates as White individuals; however, smartphones represent the only web-based access for 12% of the African American population and 22% of the Hispanic population, whereas only 4% of White individuals only access the internet via smartphones [13]. Furthermore, African American individuals experience disruptions in access, as they are twice as likely as White individuals to cancel or suspend mobile phone services because of cost [13]. These interruptions are particularly vital because African American individuals are more likely to use smartphones for web-based access than White individuals [14,15]. To use health information technology (HIT) to help support following the recommended self-management health behavior, individuals must both have access to ICTs and possess the requisite skills to use them [16,17]. African American individuals experience barriers to HIT use because of inequitable access and disparities in skills required to use technology designed to support chronic disease self-management [16]. Consequently, the extent to which this technology is effective in supporting Hispanic and African American patients for chronic disease self-management is unclear. Understanding efficacy is imperative given persistent disparities in health outcomes and in HIT access and use.

Sociocultural factors also influence individuals from ethnic minority groups’ use of consumer-oriented HIT. Trust, perceived credibility, attitudes, and perceptions predict health technology acceptance and use [17]. For example, over a decade of research describes how African American individuals have different attitudes than White individuals regarding technology innovations in health care, and these factors predict HIT acceptance [18]. Trust is an important consideration in the design of health informatics interventions to promote health and wellness [19]. Sociocultural barriers (eg, unwanted attention) are among the barriers Hispanic populations report for consumer-oriented HIT [20].

Sociocultural factors present barriers that contribute to intervention-generated inequality [21,22]. Intervention-generated inequality occurs when technology-enabled health informatics approaches disproportionally benefit most populations [17]. Therefore, these interventions are less effective for minority populations and can essentially exacerbate population disparities that contribute to health inequity [23]. HIT-enabled health promotion can be enhanced by developing HIT that considers sociocultural factors that influence use (eg, levels of health literacy and digital literacy, lack of access to, or knowledge of digital tools) [24]. Systematic reviews of consumer-oriented HIT to support health and wellness find that articles do not adequately consider sociotechnical factors [25].

### Objectives

HIT research describes the potential benefit from the use of technologies designed to track and report health behaviors, along with the acknowledgment of sparse insights to guide researchers concerning specific barriers to use for ethnically diverse populations [20]. However, no systematic review has been published describing the efficacy of consumer-oriented HIT designed to support following recommended self-management behavior for African American or Hispanic patients with chronic diseases. Therefore, we conducted this study of efficacy of consumer-oriented HIT in these patients. For this study, we classify *consumer-oriented HIT* as a technology designed to support recommended chronic disease self-management. It includes a myriad of mobile, tablet, and computer apps designed to support following recommended chronic disease self-management behaviors, such as electronic journals to track physical activity and prompts and reminders to support medication behavior. HIT also includes technology that enables access to health information, such as podcasts and disease-specific discussion boards. Given that ethnic minority populations experience both persistent inequity in chronic disease outcomes and barriers to access of consumer-oriented HIT designed to support following recommended self-management behavior, this study was guided by the following research question: what is the impact on clinical outcomes of consumer-oriented HIT interventions on self-management behavior or health outcomes for Black or Hispanic patients with chronic diseases?

## Methods

### Overview

After confirming health equity as the focus of this study, we followed an a priori protocol with equity as the focus, the PRISMA (Preferred Reporting Items for Systematic Reviews and Meta-Analyses)-Equity 2012 Extension was selected as a guideline for conducting systematic reviews that focus on health equity [26,27].

### Inclusion Criteria

We developed a rationale for eligible study designs and inclusion of outcomes, per the PRISMA-Equity 2012 Extension for systematic reviews [27]. First, we identified foundational articles based on the refined research question [28-31]. We then reviewed papers from several journals that published the foundational articles and published the foundational articles and journals that published papers that the foundational articles cited. Given the interdisciplinary nature of health equity research, we selected established journals, with an emphasis on health equity (eg, *Social Science & Medicine*, the *Journal of Racial and Ethnic Health Disparities*, and the *Journal of Health Care for the Poor and Underserved*) and from medical informatics (eg, the *Journal of American Medical Informatics Association* and the *Journal of Medical Internet Research*). We chose a systematic review based on the types of articles appearing in these journals. Given that outcomes are germane for describing inequity, we selected journals that reported outcomes.

### Information Sources

Next, we crafted themes of interest, again per the PRISMA-Equity 2012 Extension for systematic reviews [26], which formed the foundation of our inclusion and exclusion criteria: health technology designed for patients, Unified Theory of Acceptance and Use of Technology, theme (eg, acceptance, usability, readiness, satisfaction, and preference), self-management (eg, self-management behavior, health behavior, adherence, and compliance), health conditions (eg, chronic disease and physical health), and demographics.

To evaluate and select databases, we again reviewed the 4 foundational articles. We also consulted with a health sciences librarian to evaluate and finalize the databases. We selected seven electronic databases: PubMed, Cumulative Index of Nursing and Allied Health Literature, Web of Science, Cochrane, Compendex, Institute of Electrical and Electronics Engineers, and Computers and Applied Sciences Complete.

### Search Strategy

We created search strings based on our themes of interest (eg, acceptance, usability, readiness, satisfaction, and preference), according to the specific database format, to locate articles that met our inclusion criteria. We consulted with health science librarians to ensure adherence to the database string format. Information regarding the search strategy (eg, search strings) is given in Multimedia Appendix 1 [30,32-57]. When the database permitted, all results were limited to peer-reviewed journal articles published after 1990 as the World Wide Web was introduced during this period. All database searches were conducted on November 26, 2018. In addition, PJM hand-searched references of the included articles to ensure all pertinent articles were included.

### Study Selection

Articles were included if they met specific inclusion criteria and excluded if they fulfilled the exclusion criteria (Textbox 1).

Rayyan (Rayyan Inc), an internet-based software package, was used to facilitate article screening [58]. CRS and PJM blindly completed the title and abstract and full-text screening. They resolved conflicts together after the blind screening feature in Rayyan was turned off.

**Article inclusion and exclusion criteria.**

**Inclusion criteria**

- Articles included patients with chronic diseases or caregivers who specified they were of Black or African American, or Hispanic origin.
- The patient or caregiver must be the end user or direct benefactor of technology.
- Technology gives personalized information to patients and or caregivers.
- Technology was designed to support self-management recommended for chronic conditions (ie, medication behavior, physical activity, dietary behavior, and attending follow-up appointments).
- The article is in English in a peer-reviewed journal.
- The article has been published since 1990.

**Exclusion criteria**

- Intervention targets providers.
- No electronic technologies (ie, technology using electricity) examined in the article.
- Technology is not designed to support self-management recommended for chronic conditions (ie, medication behavior, physical activity, dietary behavior, and attending follow-up appointments). Technology designed to prevent falls was not included.
- A systematic review of technology.

### Data Collection Process and Data Items

Once conflicts were resolved, we analyzed the included articles and extracted relevant information (Table 1). Given the focus of our review is technology designed for chronic disease self-management for African American and Hispanic patients, we detailed information concerning race or ethnicity and cultural tailoring, type of technology used, behavior targeted, and specific chronic disease and clinical outcomes measured. We used content analysis to classify themes and totaled the frequency of self-management activities reported.

We analyzed the risk of bias in each included article using the Cochrane Collaboration Risk of Bias Tool [59]. The tool was developed in 2005 based on the following seven principles for assessing risk of bias in randomized trials: (1) avoiding use of quality scales (eg, because scales, and resulting scores, are inappropriate appraisals of clinical trials, their use increases risk of bias), (2) focusing on internal validity (eg, a small trial with high internal validity may have high risk of bias, whereas a large trial, while having high precision may have high risk of bias if internal validity is low), (3) assessing the risk of bias in trial results (eg, the quality of the reporting—which may be assessed by evaluating level of detail—helps determine the risk of bias; methodology used in conducting the trial—such as not calculating the sample size with power analysis, not including ethical review board approval, or limiting participants’ knowledge of intervention received can all increase the risk of bias), (4) using judgment when assessing risk of bias (eg, omitting bias assessments from aspects of the trail methodology or interpretation of results may increase risk of bias), (5) choosing domains to be assessed (eg, if detail is not described for how incomplete data were accounted for, or aspects of blinding for participants and practitioners, can increase the risk of bias), (6) focusing on the risk of bias in the data as represented in the article (eg, the exclusion of certain participants in trial results who are then reinstated for other results increases the risk of bias), and (7) reporting outcome-specific evaluations of the risk of bias (eg, describing randomized allocation to control or experimental group during participation may influence the risk of bias in other aspects of the trial, such as physicians’ knowledge of the specific intervention and its usual effects). The tool contains six domains for assessing potential bias, with sources of bias in each domain: (1) selection bias (inadequate generation of a randomized sequence and inadequate concealment of allocations before an assignment increase the risk of bias), (2) performance bias (inadequate blinding of participants and study personnel increases the risk of bias), (3) detection bias (inadequate blinding of outcome assessment increases the risk of bias), (4) attrition bias (incomplete outcome data for outcomes reported increases the risk of bias), (5) reporting bias (selective reporting increases the risk of bias), and (6) other bias (ie, any bias not included in the other 5 named domains).

**Table 1:** General characteristics (N=27).

Characteristics^a^			Values, n (%)
**Self-management area**			
	Medication behavior	17 (62)	
	Follow-up appointment attendance	4 (14)	
	Physical activity	17 (62)	
	Dietary behavior	16 (59)	
**Care setting**			
	Home (capability to access or use from home)	27 (100)	
	Hospital^b^	2 (7)	
**Technology**			
	Computer, laptop, or tablet^c^	3 (11)	
	Telephone (landline)	0 (0)	
	Mobile phone	17 (62)	
	Mobile app	1 (3)	
	Text	15 (55)	
	Web-based	8 (29)	
	Bluetooth device	2 (7)	
	Specialized telemedicine device	2 (7)	
	Nintendo Wii	1 (3)	
	Voice-enabled device	1 (3)	
	Social media	1 (3)	
**Function**			
	Collecting personal health data^d^	13 (48)	
	Goal setting and tracking	17 (62)	
	Integrated survey and assessment	19 (70)	

^a^Articles may be included within multiple categories.

^b^We did not include articles in which users could use videos to chat or communicate with providers.

^c^Telemedicine units or devices were included.

^d^Tracking of patient’s personal health data (data logs) and tracking of patient data by providers were included.

## Results

### Study Characteristics

**Figure 1:**
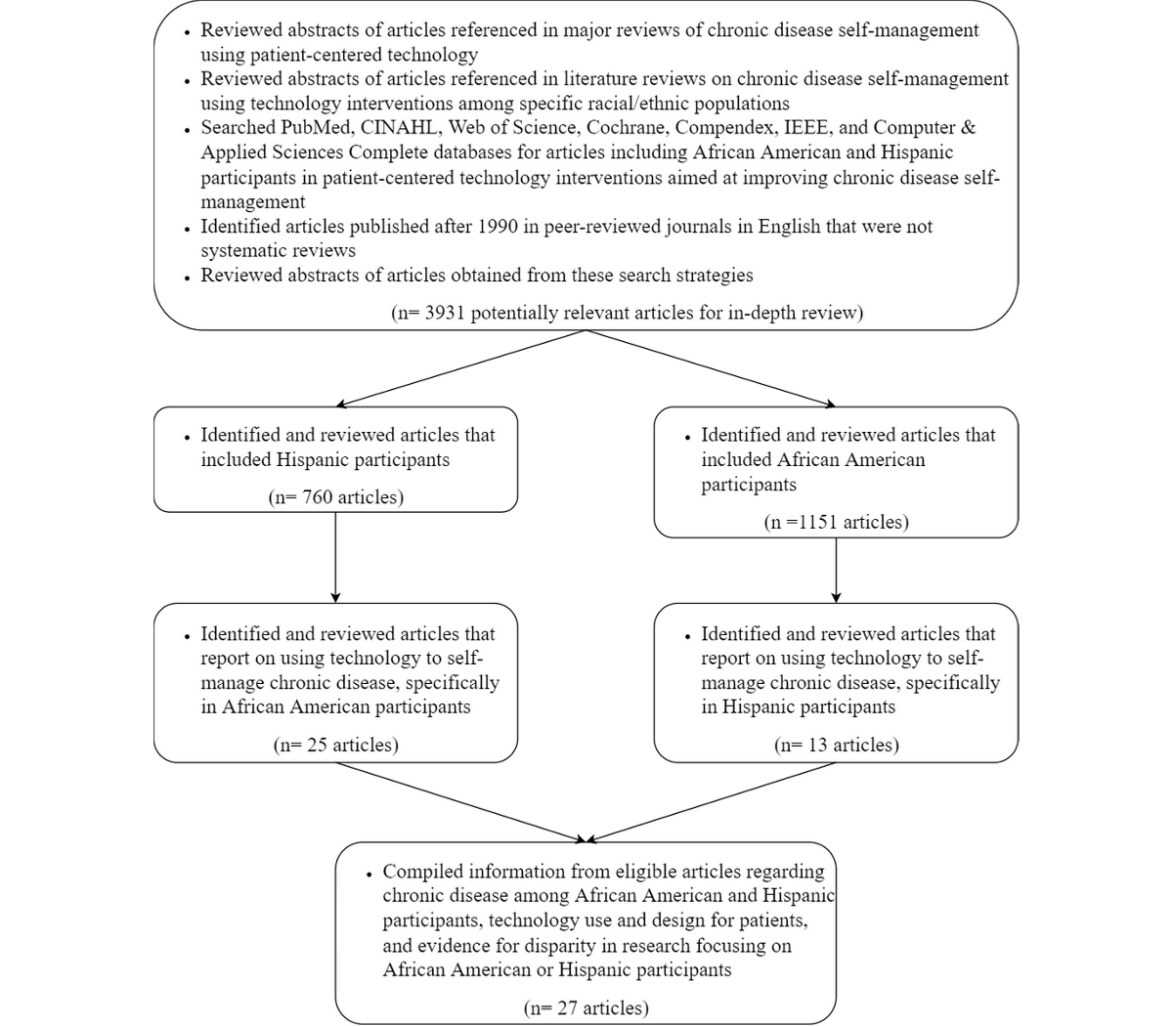
PRISMA (Preferred Reporting Items for Systematic Reviews and Meta-Analyses) flowchart.

A total of 25 eligible articles involving African American participants and 13 articles with Hispanic participants were identified. Of these, only 27 met our final criteria, as not all articles discussed technology use and design for patients (see PRISMA flowchart in Figure 1). All 27 articles were published between 1996 and 2018. The mean participant sample size was 640 (SD 209.5; 26/27, 96% of articles). Of the 27 included articles, 14 (52%) focused exclusively on African American patients, 4 (15%) focused on Hispanic patients, and 9 (33%) focused on both African American and Hispanic patients.

Each of the 27 included articles was examined for the risk of potential bias according to each of the 6 domains (Table 2).

**Table 2:** Risk of bias in individual articles (N=27).

Study	Participants, n	Patients or caregivers involved in the design of technology	Incomplete outcome data^a^	Blinding of participants or personnel^b^	Other bias^c^
Almeida et al [32]	452	High	Low	Low	Not reported
Collins and Champion [33]	15	Not reported	Low	Low	Not reported
Davidson et al [34]	50	Low	Low	Low	Low
Davis et al [35]	51	Low	Low	High	Not reported
Finkelstein et al [36]	30	Not reported	Low	Low	Not reported
Finkelstein and Wood [37]	N/A^d^	High	High	Not reported	Low
Fortmann et al [38]	414	Low	Low	Low	Not reported
Friedman et al [39]	267	Not reported	Low	Low	Not reported
Gerber et al [40]	95	Not reported	Not reported	High	High
Green et al [41]	9298	Low	Low	Low	Low
Grimes et al [42]	12	Low	Not reported	High	Not reported
Heitkemper et al [30]	220	Low	High	Not reported	Not reported
Joseph et al [43]	29	Low	Low	Low	Not reported
Kline et al [44]	123	Not reported	Low	High	Not reported
MacDonell et al [45]	48	Low	High	Low	Low
Lin et al [46]	124	High	Low	Low	High
Mayberry et al [47]	19	Low	Low	High	Not reported
McGillicuddy et al [48]	12	Low	Low	Low	Not reported
Newton et al [49]	97	Not reported	High	Low	High
Nundy et al [50]	15	Not reported	Low	Not reported	Not reported
Reese et al [51]	14	Low	High	High	Not reported
Reininger et al [52]	71	Not reported	Low	High	Not reported
Rosal et al [53]	89	Low	Low	Low	Low
Shea [54]	1665	High	High	Low	Low
Skolarus et al [55]	94	Low	High	Low	Low
Trief et al [56]	1665	Low	Low	Low	Not reported
Weinstock et al [57]	1665	Low	Low	Low	Not reported

^a^Outcome data.

^b^Randomization or blinding of patients.

^c^Any other bias identified by the reviewers.

^d^N/A: not applicable.

### Additional Analyses: Qualitative Synthesis

Articles that reported technology interventions and included self-management aimed at improving chronic disease outcomes using either clinical or behavioral outcomes were eligible for systematic review inclusion (Table 3). We chose content analysis for categorizing data into themes and counting their frequency based on our decision to count frequency of self-management behaviors [60]. We reported the following four specific self-management activities: medication behavior, physical activity, dietary behavior, and follow-up appointment attendance. The frequency of each self-management behavior was totaled by analyzing the included articles.

**Table 3:** Self-management behaviors in the included articles (N=27).

Study	Medication behavior	Follow-up appointment attendance	Physical activity	Dietary behavior
Almeida et al [32]	No	No	Yes	No
Collins and Champion [33]	No	No	Yes	Yes
Davidson et al [34]	Yes	No	No	No
Davis et al [35]	Yes	No	Yes	Yes
Finkelstein et al [36]	No	No	Yes	No
Finkelstein and Wood [37]	No	No	Yes	No
Fortmann et al [38]	Yes	No	Yes	Yes
Friedman et al [39]	Yes	No	No	No
Gerber et al [40]	No	No	Yes	Yes
Green et al [41]	Yes	Yes	No	No
Grimes et al [42]	No	No	No	Yes
Heitkemper et al [30]	Yes	No	Yes	Yes
Joseph et al [43]	No	No	Yes	No
Kline et al [44]	Yes	No	Yes	Yes
MacDonell et al [45]	Yes	No	No	No
Lin et al [46]	No	No	Yes	Yes
Mayberry et al [47]	Yes	No	Yes	Yes
McGillicuddy et al [48]	Yes	No	No	No
Newton et al [49]	No	No	Yes	Yes
Nundy et al [50]	Yes	Yes	No	Yes
Reese et al [51]	No	No	Yes	No
Reininger et al [52]	Yes	No	Yes	Yes
Rosal et al [53]	Yes	No	Yes	Yes
Shea [54]	Yes	No	No	No
Skolarus et al [55]	Yes	No	Yes	Yes
Trief et al [56]	Yes	Yes	No	Yes
Weinstock et al [57]	Yes	Yes	No	Yes

### Study Selection

Other data recorded from the articles included the technology functions (Table 4), the type of technology used, the effectiveness of the technology, the number of participants enrolled, and the first author’s last name (Multimedia Appendix 1). These were grouped together to reveal findings such as which technology was effective based on population and chronic disease type (Multimedia Appendix 1) [29,30,32-57].

**Table 4:** Technology functions in the included articles (N=27).

Study	Tracking by a patient or caregiver using technology	Tracking or viewing patient data by a patient or caregiver	Tracking of patient data by providers	Goal setting or tracking	Integrated surveys or assessments
Almeida et al [32]	No	No	Yes	Yes	No
Collins and Champion [33]	No	No	No	No	Yes
Davidson et al [34]	Yes	Yes	Yes	Yes	Yes
Davis et al [35]	No	No	No	Yes	Yes
Finkelstein et al [36]	No	Yes	Yes	Yes	No
Finkelstein and Wood [37]	No	Yes	Yes	Yes	No
Fortmann et al [38]	No	No	No	Yes	No
Friedman et al [39]	No	No	No	Yes	Yes
Gerber et al [40]	No	No	No	No	No
Green et al [41]	Yes	Yes	Yes	No	Yes
Grimes et al [42]	No	No	No	Yes	No
Heitkemper et al [30]	No	No	No	Yes	Yes
Joseph et al [43]	No	Yes	Yes	Yes	Yes
Kline et al [44]	No	No	No	No	Yes
MacDonell et al [45]	No	No	No	No	Yes
Lin et al [46]	No	Yes	Yes	Yes	No
Mayberry et al [47]	No	Yes^a^	No	Yes	Yes
McGillicuddy et al [48]	No	Yes	Yes	No	No
Newton et al [49]	No	Yes	Yes	No	Yes
Nundy et al [50]	Yes	No	Yes	No	Yes
Reese et al [51]	No	No	No	Yes	Yes
Reininger et al [52]	No	No	No	No	Yes
Rosal et al [53]	No	Yes	Yes	Yes	Yes
Shea [54]	No	Yes	Yes	No	Yes
Skolarus et al [55]	No	Yes	No	Yes	Yes
Trief et al [56]	No	Yes	Yes	Yes	Yes
Weinstock et al [57]	No	Yes	Yes	Yes	Yes
Total, n (%)	4 (14)	14 (51)	15 (55)	17 (62)	19 (70)

^a^Coaching of family members via phone was also conducted.

### Health Outcomes Described in the Included Articles

Diabetes, hypertension, and heart failure were the three chronic conditions included in the resultant studies (N=27). Diabetes was the most common chronic disease among these studies. Of the total number of studies, 8 specifically tracked hemoglobin A_1c_ (HbA_1c_) and blood pressure (BP) levels [30,35,38,44,54,56,57]. Only a study by Weinstock et al [57] reported statistical significance for both clinical outcomes. A study by Davis et al [35] reported increases in medication adherence and self-efficacy for diabetes medication behavior in African American patients. However, none of the results were statistically significant, and they did not report any clinical significance. The study by Weinstock et al [57] targeted A_1c_ reduction in Hispanic and African American patients. The intervention included a home telemedicine unit with a web-enabled camera for a videoconference consultation, which provided educational information. Results showed HbA_1c_ improvement for Hispanic patients; however, improvement was not statistically significant and clinical significance was not specified. A study by Shea [54] used a home telemedicine–specialized device for videoconferencing with a nurse to support HbA_1c_ and BP monitoring. The intervention reported clinically significant A_1c_ improvement (8.35%-7.42%), but the results were not statistically significant. A study by Heitkemper et al [30] used a website and SMS text messages for diabetes management targeting African American and Hispanic patients. Use was low among participants because they rarely used the internet to search for health information; consequently, outcomes were not reported. In a study by Kline et al [44], the intervention was a culturally tailored guide for diabetes management targeting the Hispanic population. It included a telenovela with learning modules and games. However, specific clinical outcomes (eg, A_1c_) were not reported, as the focus was on the development and feasibility of the intervention.

Hypertension was the next most common condition specified (ie, they focused on hypertension vs BP reporting). Three studies specified the goals of reducing hypertension [39,48,55]. However, none reported statistical or clinical significance. A study by Skolarus et al [55] used an SMS text messaging intervention with a faith-based collaborator and reported both systolic and diastolic BP for African American patients. Half of the participants reached BP targets. A study by McGillicuddy et al [48] used a mobile health intervention that targeted Hispanic patients to promote BP improvement through medication self-management support. The study reported statistically significant increases in medication adherence. A study by Davidson et al [34] reported statistically significant results in systolic and diastolic BP reduction in Hispanic and African American participants. The system used electronic medication dispensers and SMS text messages. It included Bluetooth-enabled BP monitors.

Heart failure was the third chronic disease that was the focus of one of the resultant studies. A study by Finkelstein and Wood [37] assessed the feasibility of an intervention that used a laptop and Nintendo Wii to support medication behavior in African American patients with heart failure. Although clinical outcomes were not reported, participants reported a high level of acceptance of the technology.

## Discussion

### Principal Findings

Given the development of HIT apps and considerable research in this area, a relatively small number of resultant articles (N=27) investigated associations between the use of HIT and chronic disease outcomes among African American and Hispanic patients. This is a vital gap because of persistent inequity in chronic disease outcomes for racial minority populations and because intention to use HIT designed for chronic disease self-management is most predicted by performance expectancy, followed by social influence [61]. Researchers of HIT acceptance and use for chronic disease self-management should incorporate health outcomes in investigations, particularly outcomes commonly used to report racial inequity. Of the 27 articles, a majority addressed 3 of the 4 self-management behaviors investigated: medication behavior (17/27, 63%), physical activity (17/27, 63%), and dietary behavior (16/27, 59%). However, only a few (4/27, 15%) focused on follow-up appointment attendance. This is an area that warrants investigation and development of capabilities because HIT may be well-positioned to mitigate known causes of missed appointments, which is a persistent issue among racial minority populations who experience persistent inequity, such as Latinx immigrants and low-income African American patients in urban settings: forgetfulness, transportation barriers, family and employer obligations, and anticipated long clinic wait times [62-64]. Investigating and alleviating barriers to appointment attendance is important because ethnic minority patients are more likely to have low income and live in urban areas, two factors that are associated with the frequency of missing primary care appointments [65-67].

All the articles investigated HIT designed with capabilities to access or use at the patient’s home, whereas only 2 articles also included use in the hospital. This is concordant with the movement of developing HIT for use in patients’ homes versus hospitals [68]. The risk of misuse of HIT is segmented according to environmental, human, and technological factors [69]. The number of different users is associated with the risk of misuse because users may have various levels of education, instruction, or training. Thus, the risk of misuse is higher in home care settings when compared with hospital care settings [68]. Developers should consider the known risks of misuse and the number of users in home care settings for African American and Hispanic patients, given that individuals from racial minority populations have different attitudes than White patients regarding technology innovations in health care, and these factors predict HIT acceptance [18].

Various technologies are included in the resultant articles, except for the landline telephone, in which none of the articles were investigated. This follows the broad trend that more than half of US households are reliant on mobile phones and do not have landlines. In addition, Hispanic and Black adults are more likely than White adults to live in households with only mobile phones [70].

The collecting and tracking of personal health data, which over 10% of users are doing on behalf of someone else (eg, caregivers), and goal setting and evaluation are pertinent capabilities that are closely related to self-management behavior [71,72]. In addition, given the association of social influence with intention to use HIT, caregivers and other members of the patient’s network should be incorporated into the design of HIT. This may be especially pertinent for Black women who report feeling responsible for providing emotional and tangible support to homebound parents who may live in the home [73]. In fact, incorporating shared tracking use should inform HIT design, and models have been created that reflect the interplay of social context and health tracking [71].

Insights derived from this study of the 27 resultant articles reveal the potential for future development and evaluation of HIT tools in two distinct areas—known barriers faced by members of ethnic minority groups in using HIT and the unique barriers they may face in following self-management recommendations. For example, in a limited sample size, a mobile phone–based intervention that combined SMS text messaging with nursing care showed improvement in following recommended self-management behavior (ie, medication behavior, glucose monitoring, foot care, physical activity, and dietary behavior) for Black adults with diabetes [74]. In addition, a diabetes self-management education intervention for medically underserved populations showed specific impact on outcomes that are characterized by racial disparities and HbA_1c_ improvement at 6 and 12 months [30].

Despite these important findings, more specific research is needed to elucidate the sociocultural factors that in particular are known to impact HIT acceptance and use [75]. For example, the level of trust is associated with HIT acceptance and use in the context of diabetes self-management [76-78]. Moreover, factors for older adults from racial minority groups should be specifically investigated because they are less likely than individuals who do not belong to racial minority groups to use health management sites and search the web for health information to support chronic disease self-management [79]. Finally, investigations of HIT acceptance, use, and impact on self-management and outcomes should be conducted with larger samples. Despite considerable literature on drivers of inequity and the emergent literature describing the potential for HIT to support chronic disease self-management, the literature suggests that persistent disparities in chronic disease outcomes are in part because of the lack of large-scale, HIT-enabled interventions that support following self-management recommendations and report impact on outcomes [75,80-82]. In addition, given the limited reporting of clinical outcomes that inform equity measures (eg, HbA_1c_), more research is needed to understand if or how access and then use may impact following recommended self-management behavior and subsequent outcomes. Doing so may reveal critical insights to associate HIT access with outcomes, particularly imperative given persistent barriers to technology acceptability and use [75].

This study has a key limitation. We only examined articles that specified Black and Hispanic users. Specific cultural factors may emerge from a broader examination, given that various cultural factors influence both technology acceptance and use (eg, practices, customs, language, and communication) [82,83]. Understanding cultural factors is essential because they can influence the way an individual interprets health information, how they define symptoms, and if and who they decide should provide them care [75]. Therefore, individuals’ sociocultural factors must be considered in the design and use of *culturally informed HIT* [84]. This insight is vital because cultural competence is specified as a critical aspect in developing technology to help reduce health inequity globally; in fact, this has become a popular concept in various countries for improving quality of care, specifically access to respectful and responsive health care [85].

### Conclusions

The proliferation of technology-enabled tools designed to support people in following recommendations for chronic disease self-management has outpaced the research describing the degree to which the Black and Hispanic populations use this technology to support self-management behavior. Although factors driving the general use among the Black and Hispanic populations continue to be investigated, little is known about their impact on health outcomes because of their use. In this paper, we have helped to address this important gap because various technology skills are required to use consumer-oriented HIT designed to support recommended self-management and doing so may require considerable effort from the patients [86]. For example, deciphering the vast and growing amount of information requires that individuals access, assess, and organize various health information. To help elucidate gaps in the literature, we conducted this systematic review to understand the extant literature concerning the use of ICTs among Hispanic and Black people to support chronic disease self-management and highlight potential gaps.

## Appendix

Multimedia Appendix 1 A list of search strings and their corresponding databases, the care setting and self-management behavior for each included article, and technology effectiveness in managing chronic disease for each included article.

## Abbreviations

BP: blood pressure
HbA_1c_: hemoglobin A_1c_
HIT: health information technology
ICT: information and communication technology
PRISMA: Preferred Reporting Items for Systematic Reviews and Meta-Analyses
