# Supplementary material for: The Efficacy of Health Information Technology in Supporting Health Equity for Black and Hispanic Patients With Chronic Diseases: Systematic Review
Source: J Med Internet Res. 2022 Apr 4;24(4):e22124. doi: 10.2196/22124 (PMC9016513; doi:10.2196/22124)
Supplement: Multimedia Appendix 1 [file jmir_v24i4e22124_app1.docx]

List of databases and search strings used.

| PubMed | CINAHL | Web of Science | Cochrane | Compendex | IEEE | CASC |
| --- | --- | --- | --- | --- | --- | --- |
| "Cellular Phone"[Mesh] OR "text messaging"[All Fields] OR "texting"[All Fields] OR "text message"[All Fields] OR "cell phone"[All Fields] OR "mobile phone"[All Fields] OR "mobile computing"[All Fields] OR "mhealth"[All Fields] OR "mobile health"[All Fields] OR "Tablet computer"[All Fields] OR "multimedia"[MeSH Terms] OR "multimedia"[All Fields] OR "social media"[All Fields] OR "Facebook"[All Fields] OR "Twitter"[All Fields] OR "personal health record"[All Fields] OR "personal health records"[All Fields] OR "health information technology"[All Fields] OR "health information technologies"[All Fields] OR "ehealth"[All Fields] OR “tablet”[All Fields] OR “pc”[All Fields] OR “personal health informatics”[All Fields] OR “consumer health informatics”[All Fields] | “cellular phone” or “cell phone” or “text messaging” or “text message” or texting or “mobile phone” or “mobile computing” or “mhealth” or “mobile health” or “tablet computer” or internet or blogging or “social media” or Facebook or Twitter or telemedicine or “personal health record” or “personal health records” or “computer systems” or television or “medical informatics” or “health information technology” or “health information” or “health information technology” or “ehealth” or “user-computer interface” or “computer user” or “IT Technology” or “information technology” or “tablet” or “pc” or “health informatics” or “personal health informatics” or “consumer health information” | Cellular Phone OR text messaging OR texting OR text message OR cell phone OR mobile phone OR mobile computing OR mhealth OR mobile health OR tablet computer OR telemedicine OR personal health record OR personal health records OR computer systems OR medical informatics OR health information technology OR health information technologies OR ehealth OR computer use OR user-computer interface OR computer user OR Facebook OR Twitter OR informational technology OR pc OR health informatics OR personal health informatics OR consumer health information OR consumer health informatics OR Health Care Quality, Access, and Evaluation OR medical informatics | Cellular phone or texting or text message or cell phone or mobile phone or mobile computing or mhealth or mobile health or “tablet computer” or “audio player” or “audiovisual aids” or “audiovisual” or “video” or “videos” or “multimedia” or “internet” or “blogging” or “social media” or “facebook” or “twitter” or “health records, personal” or “personal health record” or “personal health records” or “computer systems” or health or information technology or “health informatics” or “personal health informatics” or “consumer health information” or “consumer health informatics” | Cellular phone or text messaging or texting or text message or cell phone or mobile phone or mobile computing or mhealth or mobile health or tablet computer or multimedia or internet or blogging or social media or facebook or twitter or telemedicine or health records, personal or personal health record or personal health records or computer systems or medical informatics or health information technology or health information technologies or ehealth or computer user or IT Technology or information technology or tablet or pc or health informatics or personal health informatics or consumer health information or consumer health informatics | “cellular phone” or “cell phone” or “text messaging” or “text message” or texting or “mobile phone” or “mobile computing” or “mhealth” or “mobile health” or “tablet computer” or “audio player” or “audiovisual aids” or audiovisual or video or videos or multimedia or internet or blogging or “social media” or Facebook or Twitter or telemedicine or “personal health record” or “personal health records” or “computer systems” or television or “medical informatics” or “health information technology” or “health information” or “health information technology” or “ehealth” or “radio” or “reminder system” or “educational technology” or “user-computer interface” or “computer user” or “IT Technology” or “information technology” or “bluetooth” or “tablet” or “pc” or “health informatics” or “personal health informatics” or “consumer health information” or “Health Care Quality, Access, and Evaluation” | “cellular phone” or “cell phone” or “text messaging” or “text message” or texting or “mobile phone” or “mobile computing” or “mhealth” or “mobile health” or “tablet computer” or internet or blogging or “social media” or Facebook or Twitter or telemedicine or “personal health record” or “personal health records” or “computer systems” or television or “medical informatics” or “health information technology” or “health information” or “health information technology” or “ehealth” or “user-computer interface” or “computer user” or “IT Technology” or “information technology” or “tablet” or “pc” or “health informatics” or “personal health informatics” or “consumer health information” |
| “UTAUT”[All Fields] OR “usability”[All Fields] OR “patient readiness”[All Fields] OR “patient satisfaction”[All Fields] OR “patient preference”[All Fields] OR “technology acceptance model”[All Fields] OR “TAM”[All Fields] OR “ICTAM”[All Fields] OR “senior technology acceptance model”[All Fields] OR “senior technology acceptance and adoption model”[All Fields] OR “STAM”[All Fields] OR “Center for research and education on aging and technology enhancement”[All Fields] OR “CREATE”[All Fields] OR “GOMS”[All Fields] OR “GOMS model”[All Fields] OR “Goals, operators, methods, and selection rules”[All Fields] | “UTAUT” or “patient acceptance” or “usability” or “patient readiness” or “patient satisfaction” or “patient preference” or “user acceptance” or “technology acceptance” or “technology performance” or “technology influence” or “technology expectancy” or “technology facilitation” or “technology acceptance model” or “technology adoption model” or “TAM” or “ICTAM” or “senior technology acceptance model” or “senior technology acceptance and adoption model” or “STAM” or “Center for research and education on aging and technology enhancement” or “CREATE” or “GOMS” or “GOMS model” or “Goals, operators, methods, and selection rules” | UTAUT OR acceptance OR usability OR readiness OR satisfaction OR patient preference OR user acceptance OR technology acceptance OR patient compliance OR technology performance OR technology influence OR technology expectancy OR technology facilitation OR patient participation OR health care access OR technology acceptance model OR technology adoption model OR TAM OR ICTAM OR senior technology acceptance model OR senior technology acceptance and adoption model OR STAM OR Center for research and education on aging and technology enhancement OR CREATE OR GOMS OR GOMS model OR Goals, operators, methods, and selection rules | “UTAUT” or “patient acceptance” or “usability” or “readiness” or “patient preference” or “user acceptance” or “technology acceptance” or “technology expectancy” or “technology acceptance model” or “technology adoption model” or “TAM” or “ICTAM” or “senior technology acceptance model” or “senior technology acceptance and adoption model” or “STAM” or “Center for research and education on aging and technology enhancement” or “CREATE” or “GOMS model” | UTAUT or patient acceptance or usability or readiness or patient preference or user acceptance or technology acceptance or technology performance or technology influence or technology expectancy or technology facilitation or technology acceptance model or technology adoption model or TAM or ICTAM or senior technology acceptance model or senior technology acceptance and adoption model or STAM or Center for research and education on aging and technology enhancement or CREATE or GOMS or GOMS model or Goals, operators, methods, and selection rules | “UTAUT” or “patient acceptance” or “usability” or “patient readiness” or “patient satisfaction” or “patient preference” or “user acceptance” or “technology acceptance” or “technology performance” or “technology influence” or “technology expectancy” or “technology facilitation” or “technology acceptance model” or “technology adoption model” or “TAM” or “ICTAM” or “senior technology acceptance model” or “senior technology acceptance and adoption model” or “STAM” or “Center for research and education on aging and technology enhancement” or “CREATE” or “GOMS” or “GOMS model” or “Goals, operators, methods, and selection rules” | “UTAUT” or “patient acceptance” or “usability” or “patient readiness” or “patient satisfaction” or “patient preference” or “user acceptance” or “technology acceptance” or “technology performance” or “technology influence” or “technology expectancy” or “technology facilitation” or “technology acceptance model” or “technology adoption model” or “TAM” or “ICTAM” or “senior technology acceptance model” or “senior technology acceptance and adoption model” or “STAM” or “Center for research and education on aging and technology enhancement” or “CREATE” or “GOMS” or “GOMS model” or “Goals, operators, methods, and selection rules” |
| “Self care”[MeSH Terms] OR “Self care”[All Fields] OR “Health behavior”[MeSH Terms] OR “Health behavior”[All Fields] OR “patient adherence”[All Fields] OR “patient compliance”[All Fields] | “self care” or “health behavior” or “adherence” or “patient compliance” or “technology-mediated behavior” or “patient compliance” or “patient participation” or “patient satisfaction” or “patient preference” | Self care OR Health behavior OR adherence OR compliance OR technology-mediated behavior OR patient compliance OR patient participation OR patient satisfaction OR patient preference | “self care” or “health behavior” or “patient adherence” or “patient compliance” or “technology-mediated behavior” or “patient participation” or “patient satisfaction” or “patient preference” | self care or health behavior or patient adherence or patient compliance or technology-mediated behavior or patient participation or patient satisfaction or patient preference | “self care” or “health behavior” or “adherence” or “patient compliance” or “technology-mediated behavior” or “patient compliance” or “patient participation” or “patient satisfaction” or “patient preference” | “self care” or “health behavior” or “adherence” or “patient compliance” or “technology-mediated behavior” or “patient compliance” or “patient participation” or “patient satisfaction” or “patient preference” |
| “Chronic disease”[MeSH Terms] OR “Chronic disease”[All Fields] OR “recurrence”[MeSH Terms] OR “recurrence”[All Fields] OR “multiple chronic conditions”[All Fields] OR “multiple chronic conditions”[MeSH Terms] | “chronic disease” or “chronic conditions” or “mental health” or “physical health” or “recurrence” or “multiple chronic conditions” | Chronic disease OR chronic conditions OR mental health OR physical health OR recurrence OR multiple chronic conditions | “chronic disease” or “chronic conditions” or “mental health” or “physical health” or “recurrence” or “multiple chronic conditions” | chronic disease or mental health or physical health or recurrence or multiple chronic conditions | “chronic disease” or “chronic conditions” or “mental health” or “physical health” or “recurrence” or “multiple chronic conditions” | “chronic disease” or “chronic conditions” or “mental health” or “physical health” or “recurrence” or “multiple chronic conditions” |
| "Hispanic Americans"[Majr] OR "Hispanic Americans"[All Fields] OR "Hispanic"[All Fields] OR "Hispanics"[All Fields] OR "Spanish Americans"[All Fields] OR "Latino*"[All Fields] OR "Latina*"[All Fields] OR "Spanish speaking"[All Fields] OR "African Americans"[Majr] OR "African American"[All Fields] OR "Black"[All Fields] OR "Blacks"[All Fields] AND “Informatics”[All Fields] | “Hispanic Americans” or “Hispanic” or “Hispanics” or “Spanish Americans” or “Latina” or “Latino” or “Spanish speaking” or “African Americans” or “Black” or “Blacks” or “African American” AND “Informatics” | Hispanic Americans OR Hispanic OR Hispanics OR Spanish Americans OR Latino OR Latina OR Spanish speaking OR African Americans OR Black OR Blacks OR African American | Black Americans or Black or African Americans or blacks or Hispanic Americans or Hispanic or  Spanish Americans or  Spanish speaking and technology | Hispanic Americans or Hispanic or Hispanics or Spanish Americans or Latina or Latino or Spanish speaking or African Americans or Black or Blacks or African American and (African American or Hispanic and technology) | “Hispanic Americans” or Hispanic or Hispanics or “Spanish Americans” or Latina* or Latino* or “Spanish speaking” or “African Americans” or Black or Blacks or “African American” | “Hispanic Americans” or “Hispanic” or “Hispanics” or “Spanish Americans” or “Latina” or “Latino” or “Spanish speaking” or “African Americans” or “Black” or “Blacks” or “African American” AND “Informatics” |

Description of included studies.

| 1^st^ Author, Year | # of participants | Care setting and technology used | Self-management behaviors targeted |
| --- | --- | --- | --- |
| **Almeida, 2015** | 452 | Home, computer and telephone | Physical activity |
| **Collins, 2014** | 15 | Home, mobile phone | Physical activity and dietary behavior |
| **Davidson, 2015** | 50 | Home, electronic medication trays, mobile texting, and Bluetooth monitor | Medication behavior |
| **Davis, 2017** | 51 | Home, internet based tool | Medication behavior, physical activity, and dietary behavior |
| **Finkelstein, 2015** | 30 | Home, mobile app using web-based feedback | Physical activity |
| **Finkelstein, 2013** | N/A | Home, laptop computer and a Nintendo Wii | Physical activity |
| **Fortmann, 2017** | 414 | Home, mobile text messaging | Medication behavior, physical activity, and dietary behavior |
| **Friedman, 1996** | 267 | Home, telephone | Medication behavior |
| **Gerber, 2009** | 95 | Home, mobile phone text messaging | Physical activity and dietary behavior |
| **Green, 2011** | 9,298 | Home, EHR system | Medication behavior and attending follow-up appointments |
| **Grimes, 2008** | 12 | Home, voice-based applications | Dietary behavior |
| **Heitkemper, 2017** | 220 | Home, website and text message | Medication behavior, physical activity, and dietary behavior |
| **Joseph, 2015** | 29 | Home, Facebook and text message intervention | Physical activity |
| **Kline, 2016** | 123 | Home, telenovela and computer-based | Medication behavior, physical activity, and dietary behavior |
| **Kolmodin MacDonell, 2016** | 48 | Home, Computer combined with text messages | Medication behavior |
| **Lin, 2015** | 124 | Home, text messages | Physical activity and dietary behavior |
| **Mayberry, 2016** | 19 | Home, text messaging and phone calling | Medication behavior, physical activity, and dietary behavior |
| **McGillicuddy, 2012** | 12 | Home, mobile health system, medication tray reminders, text messaging, and Bluetooth monitor | Medication behavior |
| **Newton Jr., 2018** | 97 | Home, text messaging | No medication behavior (they did measure how the app affected it, however features of the app did not specifically include medication behaviors) but did include physical activity and dietary behavior |
| **Nundy, 2013** | 15 | Home, text messaging | Medication behavior, attending follow-up appointments, no physical activity (was measured pre and post intervention but was not a function of the tech), and dietary behavior |
| **Reese, 2017** | 14 | Home, text messaging | Physical activity |
| **Reininger, 2013** | 71 | Home, website | Medication behavior, physical activity, and dietary behavior |
| **Rosal, 2014** | 89 | Home, computer and internet-based | Medication behavior, physical activity, and dietary behavior |
| **Shea, 2007** | 1,665 | Home, telemedicine unit with video and web access | Medication behavior, glucose and blood pressure (BP) monitoring, no attending of follow-up appointments (includes provider communication though and allows for video calls with nurse) |
| **Skolarus, 2017** | 94 | Home, text messaging | Medication behavior (specified for patients taking meds), physical activity, and dietary behavior |
| **Trief, 2013** | 1,665 | Home, telemedicine unit with Internet and video access | Medication behavior, **glucose and BP monitoring**, attendance/appointment follow-up (ability to video chat with provider), and dietary behavior (tele-visits with dieticians) |
| **Weinstock, 2011** | 1,665 | Home, telemedicine unit with Internet and video access | Medication behavior, **glucose and BP monitoring**, attending follow-up appointments (ability to video chat with provider), and dietary behavior (tele-visits with dieticians) |

Effectiveness of technology and use level.

| 1^st^ Author, Year | Effectiveness of technology in managing chronic condition (high, medium, low) | Technology | Use Level^a^ |
| --- | --- | --- | --- |
| **Almeida, 2015** | High – Participants who completed the computer-based interactive personal action planning session exhibited **significant** increases in physical activity (PA) participation compared to control participants | Tailored, computer-based interactive personal action planning session and interactive voice response (IVR) telephone call to increase PA among **Hispanics and African Americans** | Some |
| **Collins, 2014** | Medium - **Content analysis** of patient interviews, **most** participants seemed to use the internet as well as social media to seek exercise information | Mobile device use for internet information regarding **exercise** and SMS to communicate with **Hispanic** adolescents | Some |
| **Davidson, 2015** | High – Revealed **statistically significant** results in **time-by-treatment interactions** indicating **significant reduction** in both systolic and diastolic blood pressure (BP) | Electronic medication trays and SMS messages both reminded **Hispanic and African Americans** subjects to monitor **BP** using Bluetooth monitors | Some |
| **Davis, 2017** | Low - Results were **not statistically significant** but adherence, self-efficacy, and number of problems **showed trends** in the expected direction | Internet based **diabetes** medication tool used by **African Americans** | Some |
| **Finkelstein, 2015** | High - Results of the study showed a **significant** impact of tailored messaging on inactivity | Mobile app using web-based feedback to reduce **inactivity** in sedentary **AA** women | Some |
| **Finkelstein, 2013** | High – **Feasibility assessment**, demonstrated **high level of acceptance** of the CHF HAT laptop and Wii systems | Laptop computer and a Nintendo Wii to deliver **chronic** **heart failure** telemedicine to **African Americans** | Heavy |
| **Fortmann, 2017** | High - **Comparative evaluation** of two mHealth approaches to elucidate how technology can be integrated **most effectively** and **efficiently** within existing nurse‐led chronic care approaches to meet the complex needs of underserved individuals | Dulce Digital‐Me, a more individualized version of Dulce Digital, a **diabetes** self‐management intervention delivered to **Hispanics** via mobile text messaging | Heavy |
| **Friedman, 1996** | High - Weekly use of an automated telephone system **improved medication adherence** and **blood pressure control** in hypertension patients | Automated telephone **African American** patient monitoring and counseling | Heavy |
| **Gerber, 2009** | High - **Seventy of seventy-three women (96%)** indicated on **study satisfaction questionnaires** that they  had read the text messages | Mobile phone text messaging to promote healthy behaviors and **weight loss** maintenance in **African Americans** | Heavy |
| **Green, 2011** | Medium - Older age, lower SES, and lower levels of education were **associated** with **decreased access** to and **willingness to participate** in a Web-based intervention to improve **hypertension** control | Comprehensive EHR system called EpicCare for **Hispanics and African Americans** | Some |
| **Grimes, 2008** | Medium - **Inductive analysis** of interviews showed. Results  characterizing how  **participants were able to craft relevant stories**  and how sharing these  stories facilitated a sense of community empowerment | EatWell, a system that allows **African Americans** individuals to use their cell phones to create voice memories describing how they have tried to **eat healthfully** in their **neighborhoods** | Some |
| **Heitkemper, 2017** | Low – **Characterized** technology use of study participants. The majority **(88%)** reported rarely or never using the Internet to find health information | Mobile **Diabetes** Detective (MoDD) website and text message features for **Hispanics** and **African Americans** | Some |
| **Joseph, 2015** | High – Findings regarding acceptability were generally positive. **93%** of participants said they were motivated to continue to be physically active at the end of the study | Facebook and text message intervention to promote physical activity in **African Americans** | Some |
| **Kline, 2016** | Medium - Achieved **goal** of creating user-friendly program that depicts culturally sensitive characters and storylines. **Viewer responses** indicated they could see behaviors recommended working for them | **Diabetes management** intervention called Sugar, Heart, and Life (SHL): A Guide to Living With Diabetes that includes an interactive telenovela and user-friendly computer-based collateral learning modules and games for **Hispanics** | Some |
| **Kolmodin MacDonell, 2016** | High - **feasible** and **acceptable** to the target population, as evidenced by  **high retention rates** and **satisfaction scores** | Computer-delivered sessions based in **Motivational Interviewing** (MI) combined with text message reminders between sessions for **African Americans** | Some |
| **Lin, 2015** | Low - **No significant weight difference** between groups at baseline. **More work** is needed to validate findings | Tailored, interactive text messages for enhancing **weight loss** among **African Americans** | Some |
| **Mayberry, 2016** | High - **Descriptive stats** and **qualitative interview analysis** showed “it appealed to users, was easy to use, and is applicable to a variety of patient and family situations.” | FAMS and REACH. REACH sends tailored text messages to users and FAMS incorporates all of the REACH features with phone coaching and ability to invite a support person to receive messages. For **Hispanics and African Americans** | Heavy |
| **McGillicuddy, 2012** | High - resulted in **significant** and **sustained** increases in **medication adherence** in both patient groups | Mobile health (mHealth) **medication and blood pressure** self-management system that was patient and provider centered for **Hispanics** | Some |
| **Newton Jr., 2018** | Medium - Automated SMS text messages were **well-received** by participants, suggesting that more enhanced mHealth technologies are a **viable option** for interventions | Intervention group attended group sessions and received automated short message service (SMS) text messages designed to reinforce **behavioral strategies** among **African Americans** | Some |
| **Nundy, 2013** | High - **observed** a **high rate of satisfaction** and preliminary **evidence of improvements** in heart failure self-management | SMS-Care. Text message communication platform **African Americans** patients enrolled prior to discharge | Heavy |
| **Reese, 2017** | High - text messaging was a **feasible** and **acceptable** strategy to promote physical activity (PA). **Future studies are needed** to study the effects on behavior change | Formative assessments to understand what information in a text message would allow **African American** female college students to feel motivated, socially supported, and have a positive body image to increase **physical activity** | Some |
| **Reininger, 2013** | Medium - **in-depth qualitative insight** into the seeking, access, and use of Web-based health information | eHealth **diabetes** prevention and control website for **Hispanics and AAs** | Some |
| **Rosal, 2014** | Low - outcomes **may** be comparable to those of face-to-face interventions. **Further research needed** | Virtual world-based intervention for **African Americans** | Some |
| **Shea, 2007** | High - diabetes case management delivered using tele-medicine **improved A1C**, with **change** from 8.35% to 7.42% within the  intervention group | Home telemedicine unit (HTU) for nurse videoconferencing, **glucose and BP monitoring**, web portal access, and educational site access for **Hispanics and African Americans** | Heavy |
| **Skolarus, 2017** | High – half of participants (N=94) **reached BP targets**. No between group differences in BP change from pre- to post- intervention (Systolic or Diastolic BP) | Reach Out. a faith-collaborative, mobile health, randomized, pilot intervention trial targeting **BP reduction**. Delivered text messages encouraging healthy dietary and physical activity for **African Americans** | Heavy |
| **Trief, 2013**  (5 year follow up of the IDEATel study described in (Shea, 2007), this study focused on self-care behavior) | High – self-reported **health behavior improved significantly** compared to usual care, but minority groups showed less improvement than Whites | Home telemedicine unit with web-enabled computer with camera for video visits for **Hispanics and African Americans** | Heavy |
| **Weinstock, 2011**  (5 year follow up of the IDEATel study described in (Shea, 2007), this study focused on glycemic control and inequity) | Medium – Although usage high across sample, Black and Hispanic participants had significantly **fewer uploads** (blood glucose, BP) than non-Hispanic Whites. **Hispanics** had the highest baseline A1C and showed the greatest improvement, but **Hispanics did not achieve target A1C levels**, whereas non-Hispanic Whites did | Home telemedicine unit with web-enabled computer with camera for video visits. Capability to upload **blood glucose and BP measurements**, as well as educational resources and patient-specific data for **Hispanics and African Americans** | Heavy |

^a^. Using criteria established by Dorr et al., 2007, level of use of technology was determined by: Heavy use, some use (50% of participants were given technology or less OR one time use among participants), or no use (neither patient nor caregiver used the technology)
